# Voice-Assisted Technology for People With Parkinson's Disease Experiencing Speech and Voice Difficulties: Co-Designing Solutions Using Design Thinking

**DOI:** 10.2196/84364

**Published:** 2026-02-04

**Authors:** Jodie Mills, George Kernohan, Katy Pedlow, Orla Duffy

**Affiliations:** 1 School of Health Sciences Faculty of Life and Health Sciences University of Ulster Belfast, Northern Ireland United Kingdom; 2 School of Nursing Faculty of Life and Health Sciences University of Ulster Belfast United Kingdom

**Keywords:** voice-assisted technology, speech and language therapy, co-design, participatory methods, dysarthria, speech and voice, Parkinson's Disease

## Abstract

**Background:**

While smart speakers are emerging as a novel health care technology, people with Parkinson's Disease (PwPD) and speech and language therapists (SaLTs) have reported difficulties using smart speakers with speech and voice impairments in research. To date, PwPD have identified frustration with having to repeat themselves to be understood, devices timing out before they had finished speaking, and being unable to have a conversation with smart speakers. SaLTs have reported technical and practical challenges in implementing voice-assisted technology tools. Both PwPD and SaLTs indicated a lack of knowledge about what smart speakers could do, as well as concerns about privacy and the listening nature of the devices.

**Objective:**

This study aims to co-design solutions that support the use of smart speakers for speech and voice difficulties experienced by PwPD.

**Methods:**

Based on the Design Thinking framework, a multistage design process was conducted, involving a lay steering group and 2 online co-design workshops. Twenty participants, including PwPD, carers, SaLTs, design and technology experts, and third-sector staff, collaborated during the co-design workshops. The ideate phase included brainstorming and ranking, and conventional content analysis was used to specify prototypes.

**Results:**

Two main prototypes were created: (1) education and guidance, including privacy and therapeutic usage guides for PwPD and SaLTs to address troubleshooting and delivery considerations; and (2) new speech and language therapy (SLT)–specific features for smart speakers. Participants provided feedback on their experiences of co-design, highlighting feeling valued, the balance of perspectives, and making improvement suggestions. Feedback aligned with the UK standards for public involvement.

**Conclusions:**

Smart speakers could enhance accessibility, therapy engagement, and long-term speech outcomes, offering scalable, cost-effective solutions to support SLT services, patient independence, and reduced service demand. Smart speaker solutions with a SLT focus enable PwPD to self-manage speech and voice difficulties at home and reinforce therapy gains between clinic visits. Co-designed with users, these prototypes are intended to address health disparities and relieve pressure on SLT services, offering a scalable and sustainable solution that enhances efficiency and supports ongoing rehabilitation within health care systems.

## Introduction

### Background

Voice-assisted technology (VAT) is defined as a device that uses natural language processing or automatic speech recognition (ASR) to interpret spoken language and translate it into actionable requests.

Smart speakers are commercially available VAT devices that are controlled using voice commands and are usually connected to the internet (current examples include Amazon Alexa and Google Assistant). They can feature built-in control systems for tasks on demand, including smart home automation, providing general information (not limited to weather, recipes, or health information), person-to-person calls, sending and receiving messages, and playing music. New models with screens can also support audio and video streaming. Smart speakers are readily available for purchase and use by the general public [1].

It has been reported that VAT prompts some participants with speech difficulties to modify their speech to enable interaction with VAT [2-6]. People with Parkinson’s disease (PwPD) have reported adapting their speech by speaking more slowly, loudly, and clearly when interacting with a smart speaker [4]. Considering that 90% of PwPD present with reduced speech intelligibility and limited vocal loudness [7], VAT may hold potential as a therapeutic adjunct in speech and language therapy (SLT). This prior evidence indicates that VAT may enhance access to therapy [8].

Some therapists have reported using VAT to promote improved volume, clarity, and intelligibility of speech [9]. In addition to offering biofeedback on speech clarity, these tools have provided structured opportunities for home-based practice, fostering self-awareness and supporting the self-management of dysarthria and other speech difficulties [9,10]. PwPD have reported increased clarity of speech and volume when using VAT, and have used VAT as a communication partner to practice their speech and rebuild confidence in using their voice [11]. Both speech and language therapists (SaLTs) and PwPD agree that the objective nature of VAT is key to promoting interaction and providing feedback on speech. Textbox 1 presents a hypothetical vignette illustrating how a person with speech or voice difficulties may interact with smart speakers. This vignette is informed by the understanding and findings of previous research [11].

**Case study**

1. Case

John is 65 and has had Parkinson’s disease for 5 years. His phonation is impacted by poor breath support, resulting in a breathy, hoarse voice with low volume. His articulation is reduced, resulting in imprecise speech production, which reduces speech clarity and intelligibility. His speech is also hypernasal, and nasal emissions are noted. He has hypokinetic dysarthria. At home, his family can understand him, but he is frequently told that they “can’t hear him” and that he “needs to speak up.” This is frustrating for John, as he reports that “he feels like he is shouting,” which suggests impaired self-awareness of his speech.

2. Use

John uses his smart speaker daily. When he speaks to the smart speaker, it replies with “Sorry, I didn’t get that” approximately 50% of the time. As a result, John raises his volume and repeats his request. Often, he uses a loud voice, overarticulates his words, slows down, and speaks as soon as he takes a breath. The smart speaker responds when he uses these strategies. This demonstrates that smart speakers provide feedback on volume and clarity of speech in the form of an external cue: “Sorry, I didn’t get that.” This can encourage increased self-awareness of speech volume and intelligibility, and result in the use of LOUD, clear speech strategies. As John’s smart speaker can time out before he has finished speaking, he uses *adaptive listening mode* (available on Amazon devices), which is found in the accessibility settings and gives him longer to speak.

John also plays the game “Word Tennis” on his smart speaker. He has to think of words within a semantic category quickly and remember to use a LOUD, clear voice when answering. This task focuses on a word-level activity within the speech hierarchy and adds a cognitive load to increase difficulty, which aligns with Lee Silverman Voice Treatment (LSVT) LOUD principles [12]. He also enjoys sport and cooking and uses his smart speaker to search for recipes. Common functional requests include “Add cheese and potatoes to my shopping list,” “Show me my cooking library,” and “What was the Man United score today?” Sometimes, he even uses his smart speaker like a diary: “Leave a sticky note for...”, where he records a voice note on his smart speaker to remind someone to feed their dog.

3. Summary

Overall, interacting with his smart speaker allows John to practice a LOUD, clear voice at home, with external feedback on speech volume and clarity that may help improve his self-awareness.

Despite the facilitators discussed in Textbox 1, several barriers to the effective use of VAT among PwPD and SaLTs remain [9,11]. For example, PwPD have reported feeling frustrated by needing to repeat themselves to be understood, by devices timing out before they had finished speaking, and by being unable to have a conversation with their smart speaker [11]. SaLTs also indicated that they faced technical and practical challenges in implementing VAT tools [9]. Both PwPD and SaLTs reported a lack of knowledge about smart speaker capabilities and concerns surrounding privacy and data security.

**Figure 1:**
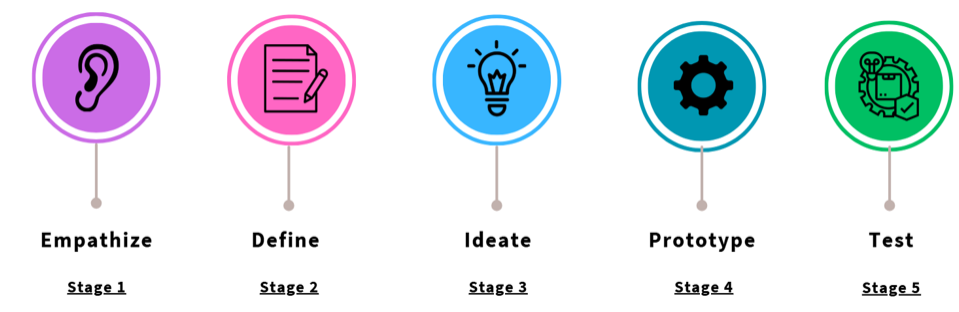
The Design Thinking stages [13] from empathize to prototyping, which can be used during the development of new health care technologies [15].

Addressing these challenges is essential to enable the integration of VAT into SLT practice. Design Thinking is a user-centered innovation framework used to guide the development of new health care technologies, often utilizing co-design approaches [13-15]. It offers a structured approach to identifying problems and generating solutions through empathy, collaboration, and iterative prototyping and testing. This study is informed by the define, ideate, and prototyping phases of the Design Thinking process. Figure 1 outlines the Design Thinking process, and Table 1 shows the connections between the phases of the Design Thinking framework, the specific research questions to be addressed, and the methods used.

Co-design has been used to foster collaboration that stimulates new ideas, clarifies concepts, and creates solutions that prioritize the needs and lived experiences of end users [16]. Co-design workshops have been used in SLT, health technology research, and with older adult populations [17-19], with improved outcomes for technology adoption compared with noncollaborative design processes [17,19]. Co-design is critical when developing technologies for SLT [18] and has value in engaging people with communication difficulties [20-22]. We set out to follow the co-design cycle and principles, meeting the criteria for true co-design under the ladder of co-production [23-25], through the identification and development of recommendations from participants with communication difficulties [26,27].

**Table 1:** Ways in which existing barriers to the therapeutic use of VAT^a^ by people with Parkinson’s can be solved.

Objective	Objective 1: To consider and select problem statements from the perspectives of experts by experience.	Objective 2: To create solutions to problem statements associated with VAT usage, alongside people with Parkinson’s, carers, SaLTs^b^, technology and design experts, and third-sector representatives.	Objective 3: To prioritize co-designed solutions to inform prototype VAT interventions.	Objective 3: To prioritize co-designed solutions to inform prototype VAT interventions.
Design Thinking stage	Stage 2: Define	Stage 3: Ideate	Stage 3: Ideate	Stage 4: Prototype
Method	Patient, public involvement workshop	Co-design workshop A	Co-design workshop B	Inductive content analysis

^a^VAT: voice-assisted technology.

^b^SaLT: speech and language therapist.

This research is intended to address previously noted barriers to VAT use [9,11] and aims to co-design solutions to previously identified challenges with VAT by working with PwPD, carers, SaLTs, charity representatives, and technology and design experts. This approach was taken to ensure that new technologies can be used in ways that meet end user needs. We sought to create solutions by using commercial technology, without coding or modifying VAT devices. This ensures that solutions are low cost and accessible, enhancing the potential for wider adoption of VAT in SLT contexts.

### Aim

We set out to co-design solutions to support the use of smart speakers in SLT to improve volume and intelligibility for PwPD, using a Design Thinking framework. The research addressed the following question: “How can we facilitate the therapeutic use of VAT by people with Parkinson's Disease?”

Our study objectives (mapped onto Design Thinking stages) are as follows:

- To consider and select problem statements from the perspectives of experts by experience (Define).
- To co-create solutions to problem statements associated with VAT usage, alongside PwPD, carers, SaLTs, technology and design experts, and third-sector representatives (Ideate).
- To prioritize co-designed solutions to inform prototype VAT interventions (Prototype).

## Methods

### Participatory Co-Design Approach

Participatory methods such as co-design allow interventions to be designed around end user needs. This study co-designed solutions to previously identified barriers regarding the use of VAT when speech and voice difficulties were present. This results in technology that more readily meets user needs [28] and helps to avoid digital exclusion [29]. Workshops were held online, removing geographical and physical barriers and enabling SaLTs from throughout the United Kingdom to share their experiences.

### Ethical Considerations

Ethical approval was granted by the Ulster University Research Ethics Committee in January 2025 (approval number FCNUR-24-078-A). This study is part of a larger PhD project using Design Thinking. Previous phases of work aligned with the empathize stage, and the current co-design phase aligns with the define, ideation, and prototyping stages. All participants provided informed consent before the workshops. Participant outputs were anonymous, and ground rules were agreed upon to maintain confidentiality. Participants did not receive payment or financial incentives.

### Patient and Public Involvement

A patient and public involvement (PPI) steering group was established to provide a voice for key stakeholders and ensure their active role in shaping the research. This group included a SaLT with firsthand experience using VAT in clinical settings, a person living with Parkinson’s, and a caregiver. These 3 experts by experience coassessed the barriers to VAT usage identified in previous research [9,11] and co-decided the top 5 problems that reflected their experiences, in keeping with the cycle of coproduction [24].

### Study Recruitment

PwPD and carers were recruited via a third-sector organization (Parkinson’s UK) using advertisements on the Parkinson’s UK research portal, Research Support Network monthly emails, and flyers at local Parkinson’s support groups in Northern Ireland. SaLTs were recruited through the Royal College of Speech and Language Therapists, including the Parkinson’s Clinical Excellence Network. In addition, Parkinson’s UK staff and technology or design experts were recruited through the lead author’s (JM) professional network. Some participants had established a relationship with the lead researcher through work with the local branch of Parkinson’s UK in Northern Ireland.

Previous research was used to determine the number of participant collaborators invited to share their experiences during the workshops (n=20) [30,31]. Participants were asked to contact the research team to express interest in the study and were screened according to predefined criteria (Table 2). Potential participants were sent study information and consent forms by email or post, depending on preference, and were asked to indicate their availability. Once consent was obtained, participants completed a demographic survey and received links for the online workshops. This enabled interaction between diverse experiences. Participants were placed into smaller, experience-diverse groups of 4-5 participants to encourage idea generation in a safe and supportive environment.

**Table 2:** Inclusion and exclusion criteria for people with Parkinson’s, carers, speech and language therapists, third-sector representatives, and technology or design experts.

Participant group	Inclusion	Exclusion
People with Parkinson’s	Adults over 18 years old Mild to moderate dysarthria/voice difficulties (to include users of augmentative, alternative communication) Diagnosis of Parkinson’s disease Current or previous use of VAT^a^ Have access to a laptop, with a camera, that facilitates videoconferencing software	Moderate or severe cognitive impairment History of other neurological disorders
Carers	Adults over 18 years old Live with or care for a PwPD^c^ or both Experience of facilitating the use of VAT with a PwPD Have access to a laptop, with a camera, that facilitates videoconferencing software	N/A^b^
Speech and language therapists	Adults over 18 years old Who currently have/have had a clinical caseload of PwPD in the past 5 years Who have used VAT in practice and have basic knowledge of the devices Have a laptop, with a camera, that facilitates videoconferencing software	N/A
Third-sector staff	Adults over 18 years old Currently working in a third-sector organization for PwPD Involvement and relationships with the local Parkinson’s community Basic knowledge of speech and voice difficulties in Parkinson’s disease Have a laptop, with a camera, that facilitates videoconferencing software	N/A
Technology/design experts	Adults over 18 years old Experience of VAT and detailed knowledge of its capabilities or relevant experience in designing or developing health care technologies	N/A

^a^VAT: voice-assisted technology.

^b^N/A: not applicable.

^c^PwPD: people with Parkinson’s disease.

### Procedure

#### Overview

**Figure 2:**
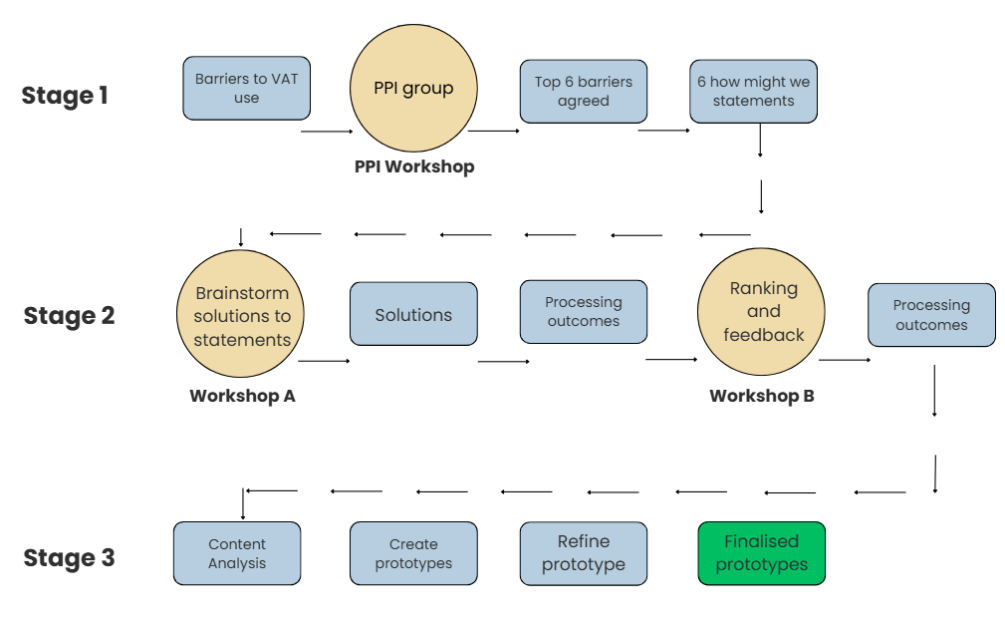
The co-design process from the development of problem statements, through workshop completion, analysis, prototype creation, and refinement. PPI: patient and public involvement; VAT: voice-assisted technology.

Using principles underpinned by Design Thinking and Participatory methodology, a series of 2 co-design workshops were undertaken, informed by insights gained from previous research [30-32]. The 3-stage co-design procedure is outlined below (see Figure 2) and aligns with the define, ideate, and prototype phases of the Design Thinking framework, as presented in Figure 1.

#### Stage 1: Define Phase

Barriers identified by PwPD, their carers, and SaLTs during an earlier stage of the research [9,11] were reviewed by the PPI group, ensuring that the research was shaped by lived experience. Researchers JM and OD were present during the workshop. The group identified and agreed on the top 6 problems to be brought forward to workshop A, and, as per Design Thinking guidance, these were reframed into “How Might We” statements. These “How Might We” statements are used in Design Thinking to help people reframe a problem-focused perspective into solution-focused thinking [33], and previous research highlights the value of shaping research based on lived experience, as this ensures that the problems being solved are meaningful to end users [34]. The final 6 “How Might We” statements are presented in Table 3.

**Table 3:** Procedures and outcomes for stages 1, 2, and 3 of the co-design process.

Stage	Design Thinking framework stage	Procedure		Outcomes
1	Define	**PPI^a^ workshop**		
			The PPI group was presented with all barriers from previous research [9,11]. JM shared her screen via videoconferencing, using Canva PowerPoint software to present the barriers identified in previous research and to assist with live decision-making. This acted as contemporaneous notes. OD also took field notes. PPI group identified 6 top problems—felt to be a reasonable number of barriers to brainstorm solutions to within an hour. 6 problems reframed into “How Might We” statements.	The following “How Might We” statements were used in workshop A: How might we help people understand smart speaker privacy and reduce their fears? How might we help people when smart speakers do not work? How might we help people to have a conversation with a smart speaker? How might we help people to know what smart speakers can do? How could we deliver this information? How could smart speaker technology be adapted?
2a	Ideate	**Co-design workshop A**		
			A document with problem statements and examples of the problems was emailed to the participants before the workshop. Welcome and introduction PowerPoint, which presented an overview of previous research, problems experienced by people using smart speakers, and an overview of the co-design process. Two example brainstorming activities completed. Smaller groups discussed 6 problem statements, rotating after 10 minutes on each problem statement. All groups completed different problems at the same time. Facilitators shaped discussions and noted contributions on a live Word document. Each problem statement had a separate Word document. Participants were invited to share solutions to the problem statements around using VAT^b^ with a speech or voice difficulty. Participants were thanked for their time and the workshop ended.	Solutions to each problem statement were reviewed. Similar solutions for each problem statement were grouped together and combined. No content was removed, while ensuring there was a feasible number of ideas to rank. Ideas are presented fully in Multimedia Appendix 1, as they appeared during the workshop. A reduced number of solutions were placed into tables for each group to rank in workshop B.
2b	Ideate	**Co-design workshop B**		
			Welcome and recap of solutions generated in workshop A. Participants received a document of the solutions ahead of the workshop to aid their preparation. Each facilitator worked through 3 solutions documents with their group and ranked their top 5 priorities for each solution. A similar procedure to workshop A was followed: each solution was captured in a separate Word document, and groups rotated after 10 minutes on each problem statement, inputting to the same solution document. Each participant was asked a short series of questions about their experience of the co-design workshops. Questions were based on the UK standards for public involvement. Participants were thanked for time, next steps were explained, and the workshop ended.	Lead researcher (JM) collated solutions and rankings and removed any solutions that were not ranked. Solutions ranked and their rankings taken forward to stage 3 (prototyping).
3	Prototyping	**5 stages of inductive content analysis conducted for solutions and rankings from workshop B**		
			Initial and final codes were discussed for refinement and agreement with the research team and broadly grouped into themes. Themes were mapped into 2 prototypes.	Two prototypes were created, namely, prototype 1 (education and guidance) and prototype 2 (developing new SLTc-specific features for smart speakers). An overview of the link between workshop 2 outputs and final prototypes is available in Multimedia Appendix 2. Participant feedback was collated.

^a^PPI: patient and public involvement.

^b^VAT: voice-assisted technology.

^c^SLT: speech and language therapy.

#### Stage 2: Ideate Phase

Co-design workshops were facilitated by 2 qualified SaLTs (JM and OD), 2 health care professionals (KP and RB), and an academic (GK), with participants working in smaller groups during the workshops. Facilitator guidance and training before the workshops ensured methodological consistency when participants worked within the groups (Multimedia Appendix 3). Co-design principles of valuing lived experience, sharing power, and respect were presented at the beginning of each workshop, aiming to reduce power imbalances between researchers and participants. Both workshops lasted approximately 1 hour and were conducted via videoconferencing to enable data collection across a wider geographical area [35], avoid travel, and facilitate workshops in the evenings.

Data collection was recorded as notes in live Word (Microsoft Corporation) documents. By recording content-only contributions, participant anonymity was ensured from the outset, as no identifiers were associated with the contributions. Although participants may have been known to other group members, they were asked to respect everyone’s right to confidentiality by not sharing contributions outside the group setting. Additionally, workshops were not audio- or video-recorded, in keeping with co-design principles. Although this may have contributed to some data loss, the live recording of workshop contributions helped to mitigate this risk.

In workshop A, participants brainstormed solutions to the “How Might We” statements shown above. The process for this workshop is shown in Table 3. Following workshop A, these solutions were refined by combining similar ideas and removing duplicates (Multimedia Appendix 1) in preparation for workshop B. In workshop B, participants reviewed the solutions to the 6 problem statements created during workshop A and were asked to rank the top solutions for each problem statement from 1 (top priority) to 5 (lower priority). Ranking is regarded as a way to prioritize and reach an agreement [36]. To ensure priorities accurately reflected participants’ lived experience, each problem statement was ranked by 2 groups; however, this meant that not every group ranked every problem statement. The workshop procedure is outlined in Table 3, and the solutions are presented in the “Results” section. At the conclusion of the workshop, participants were asked by facilitators to provide feedback on their experiences of the co-design process. Following workshop B, the lead researcher (JM) collated the rankings and removed any solutions that were not ranked.

#### Stage 3: Prototyping

Outcomes from the workshops were analyzed using content analysis and used to create prototypes, in line with the Design Thinking process. Conventional inductive content analysis, following 5 stages, was used to allow categories to emerge directly from the workshop outputs and to reduce the volume of information [37].

The lead researcher (JM) read through the workshop outputs and any associated field notes several times to become immersed in the data. This supported note-making on initial ideas in a reflexive journal, allowing consideration of connections, similarities, and differences within the data. This process also highlighted that participants generally lacked knowledge about smart speakers, were fearful of hackers, and wanted speech-accessible smart speakers. These insights challenged the lead researcher’s confirmation bias, encouraging empathy with the experiences of PwPD and allowing the research to be shaped by user needs. This highlighted the importance of creating new features for smart speakers that better meet users’ needs, as well as utilizing existing features. Reflexivity also allowed the lead researcher to reflect on her multiple roles as a SaLT, facilitator, and analyst, and the potential for these roles to introduce interpretation bias toward a clinical perspective. As a result, an audit trail was developed to demonstrate the analysis process and enhance trust in decision-making during analysis [38].

Following data immersion, the lead author created a mind map to inductively group ideas and develop initial codes for analysis. Initial codes were shared with OD, KP, and GK for discussion and refinement, enhancing credibility through investigator triangulation and peer debriefing. The final codes were both descriptive (eg, “privacy concern”) and interpretative (eg, “need to increase motivation for speech practice”), and the meaning of each code was documented to ensure reliability during coding. Subsequently, the workshop outputs were coded. This was conducted by hand, using graph paper and colored pens to assign meaning to each output.

Coding was conducted 3 times on separate days by 1 coder (JM) and was presented to the research team for discussion, redrafting, and agreement. It is acknowledged that coding by a single researcher may introduce bias, and, upon reflection, the involvement of 2 coders may have enabled data triangulation and enhanced data credibility. Despite this, peer debriefing helped to minimize potential impacts on the analysis of results. Similar codes were grouped into broader themes to capture meaning across the outputs. This process was also completed by hand, using colored pens to illustrate relationships between codes. Reflexive notes were recorded, discussed with coauthors (OD, KP, and GK), and refined accordingly. Finally, themes were conceptually mapped into 2 prototypes, in keeping with the Design Thinking Framework.

The content analysis process described above, from ranked ideas to the creation of prototypes, is available in Multimedia Appendix 2 as an audit trail, enhancing the credibility of the outputs [39]. All solutions that were ranked in workshop B were included in the prototypes to ensure that the prototypes reflected the wants and needs of participants. Furthermore, direct quotations, where available, from participant feedback are presented to provide a direct voice and to link outputs with interpretations [32]. Findings were sent to all participants for member checking to ensure that the written findings reflected their lived experiences and to enhance the rigor of the research.

## Results

### Study Participants

A total of 20 participants were recruited; 19 participated in co-design workshop A, and 16 in co-design workshop B. Overall, 15 participants took part in both workshops (Tables 4 and 5).

**Table 4:** Makeup of breakout rooms in workshop A.

Group number	Number of participants, n	Participants					
		People with Parkinson’s disease	Carer	Speech and language therapist	Third sector	Technology/design	
1	4	✓	✓	✓	N/A^a^	✓	
2	3	✓	✓	✓	N/A	N/A	
3	4	✓	✓	✓	✓	N/A	
4	4	✓	✓	✓	N/A	✓	
5	4	✓	N/A	✓	N/A	✓	

^a^N/A: not applicable.

**Table 5:** Makeup of breakout rooms in workshop B.

Group number	Facilitator	Number of participants	Participants				
			People with Parkinson’s disease	Carer	Speech and language therapist	Third sector	Technology/design
1	GK	4	✓	✓	✓	N/A^a^	✓
2	OD	4	✓	✓	✓	N/A	N/A
3	JM	4	✓	✓	✓	✓	N/A
4	KP	4	✓	N/A	✓	✓	✓

^a^N/A: not applicable.

The ranking of solutions aligned with the 6 problem statements is outlined in Multimedia Appendix 4. For problem statements 1, 2, 4, and 6, 3 solutions were ranked by both groups, and 4 solutions were ranked by 1 group. For problem statement 3, 2 solutions were ranked by both groups, and 5 solutions were ranked by 1 group. For problem statement 5, all 4 solutions were ranked by both groups, as only 4 options were presented. Solutions that were not given a rank by any group were removed from the results presented below. The full list of ideas available for ranking during workshop B is provided in Multimedia Appendix 1.

### Stage 1: Prototyping Results

Rankings were collated into 2 main prototypes by the primary researcher and agreed upon by the team: (1) educational guidance on the therapeutic use of smart speakers, and (2) developing new SLT-specific features for smart speakers (Multimedia Appendices 5 and 6). These prototypes present the results outlined above, emphasizing cross-cutting themes. Participants’ experiences of the co-design process are also presented. The process from solutions to prototypes is fully detailed in Multimedia Appendix 2.

### Prototype 1: Education and Guidance

Guides for PwPD and SaLTs, detailing how to use smart speakers to improve volume, intelligibility, and clarity of speech, were unanimously agreed upon by participants. The contents of these guides are described in Multimedia Appendix 5. Participants highlighted a gap in knowledge between the traditionally available features of smart speakers and an understanding of how these features could be repurposed to benefit speech and voice in Parkinson’s disease. The suggested skills catalog for therapy would create a repository of standard smart speaker features and skills that could be utilized with therapeutic intent by SaLTs and PwPD. Suggestions included integrating prompts and positive reinforcement by building routines, for example: “Could you speak louder?” or “Well done, great practice today.” PwPD felt that verbal prompts to speak louder or clearer, along with positive reinforcement from smart speakers, would replicate cuing provided by SaLTs during direct therapy and motivate home practice.

Participants indicated that routines could be used to practice scripted conversations, and that these should be personalized, include prompts to help sustain conversations, and contain only personal information that users felt comfortable sharing with their smart speaker.

It was evident that not all participants were aware of these accessibility features, which are designed to maximize engagement with smart speakers, and that education in this area may help to encourage more natural conversational reciprocity. For example, the conversation mode available on Amazon Alexa devices.

Participants also indicated that education about privacy relating to smart speaker use was required. It was reported that education for both PwPD and SaLTs would help to alleviate fears regarding personal data storage and General Data Protection Regulation (GDPR) concerns.

### Prototype 2: Developing New SLT-Specific Features for Smart Speakers

Participants indicated that an Alexa skill could be created to support speech therapy, as shown in Multimedia Appendix 6. Suggestions included delivering LSVT through a smart speaker or developing a speech therapy game to support speech and voice practice. Participants suggested that this could include increased feedback, such as visual cues on a screen for volume and speech clarity and live transcription of speech that repeats back what was heard, to support self-awareness in PwPD. It is acknowledged that newer Amazon Alexa models, such as the Echo Show 10, already offer subtitling features within the settings, which provide live captioning of speech or video calls.

Additionally, participants were excited about the potential for artificial intelligence (AI) integration within smart speakers and suggested that this could be used to enable more intelligent conversations with the device. Many participants indicated that current smart speakers lacked this capability. Although intelligent conversation has not yet been integrated as a core functionality across Amazon Alexa devices, skills such as ChatGPT were perceived to facilitate live, functional conversation. Furthermore, Alexa Plus, a paid feature for Amazon devices, uses generative AI to remember previous interactions and continue conversations over time. It also offers 5 personalities, which may help users feel as though they are conversing with a person rather than a device. However, Alexa Plus is not yet available in Northern Ireland, where this research was conducted. Additionally, the *follow-up* mode within Alexa accessibility settings prevents users from having to repeat the device wake word, which Amazon suggests supports a more conversational interaction with smart speakers.

Participants also indicated that extended listening time for smart speakers would prevent mid-sentence interruptions. It is acknowledged that Amazon Alexa devices currently offer an *adaptive listening* feature in the accessibility settings, which extends input time and accommodates speech differences. Although a few participants were aware of this feature, they did not indicate its impact on their smart speaker interactions.

Furthermore, enhanced privacy features were suggested. Again, it is understood that, under Alexa privacy settings, voice commands can be enabled to clear Alexa voice history; for example, “Alexa, delete everything I’ve ever said.” Additionally, although Alexa cannot be trained to respond only to certain voices, there is an option to set up a voice profile to receive more personalized content and prevent unauthorized voice purchases.

Although participants acknowledged that adapting smart speakers to better recognize dysarthric speech could hamper their therapeutic value, they felt that this would improve accessibility for the devices more generally. They sought devices that could gradually learn their speech patterns over time, as well as deal effectively with regional accents. Notably, there is currently no research exploring the impact of improved speech recognition in smart speakers on therapy outcomes in SLT.

In addition to ranking solutions, participants were asked about their experience of co-design using questions based on the UK standards for public involvement. Overall, participants valued the online workshop format, which facilitated engagement for those with limited mobility. They felt the workshops were informal yet professional and found the tasks interesting, positively challenging them to think of solutions. Small groups were reportedly the right size for supported discussions, and participants felt this was an effective way to gather substantial information. Participants discussed their expectations and involvement in co-design, describing feeling included and respected:

I had some experience of delivering co-design, so I had an idea of how it should be done...I felt valued, and felt everyone has been really equally valued, no matter how you’re coming at it; person with Parkinson’s, speech therapist, whatever. We’ve all been treated equally, with respect.

Person from Parkinson’s UK

I felt heard and respected throughout and you did a good job of facilitating conversations for us to feel heard.

SaLT

The carer and patient are heard. So often in NHS setting they are the last ones to be heard y’know, what would they know. But here, they were put front and centre.

Carer

Participants also felt that the right people were involved in the co-design process and that there was a good balance between perspectives:

It’s involved so many stakeholders that come from that same place of making improvements for people living with Parkinson’s. It was great to see various individuals are spoken to and included.

PwPD

There was a really good balance of people from different backgrounds...It absolutely worked and its so important to get everyone's view; it’s mostly important to hear people with Parkinson’s, carers you work alongside. You get a really holistic picture of what is the most important thing from different perspectives.

SaLT

It was useful to be able to discuss together in a group and helpful to consider all views: SaLTs, patients and tech experts.

PwPD

Participants provided feedback on engagement challenges and future improvements. Some PwPD or carers felt that a bridging workshop between creating and ranking solutions would be helpful. This could have included a session to discuss all brainstormed solutions and integrate them with real-world examples. Although elements of this were included in the workshops, they felt that a third workshop would have given them time to digest the large number of solutions and some more complex ideas before ranking them. One clinician who was unable to attend the first brainstorming workshop felt that this would have helped orient her more fully before the ranking task. Others suggested that color grouping or collapsing solutions for each problem statement by themes may have made it easier to rank statements. Participants also indicated that more prompts were required to remind them to think creatively and that “anything was possible.”

Participants also discussed the project’s focus on smart speakers, as well as their advantages and disadvantages. A few participants felt that it would be easier to create an app, as many are available for smartphones, and most people use these devices. However, most participants felt that the voice interaction of smart speakers offered advantages over smartphones, particularly for people with a tremor. Additionally, participants felt that smart speakers could remind and motivate users to practice, whereas with an app, users often have to self-motivate or remind themselves.

## Discussion

### Principal Findings

This study aimed to co-produce solutions to support smart speaker use for speech and voice difficulties and to inform a future intervention. PwPD, carers, SaLTs, Parkinson’s UK staff, and technology and design experts collaborated during 2 online co-design workshops to brainstorm and prioritize solutions to problems identified in prior research [9,11]. Two prototypes were developed: (1) education and guidance on the therapeutic use of smart speakers and (2) the development of new speech therapy–specific features for smart speakers. By incorporating collaborators’ priorities and needs, the study offers a foundation for a future smart speaker–based intervention for speech and voice therapy in PwPD.

### Impact of Co-Design

This project recognizes the need to involve end users early and meaningfully when designing health care interventions [40,41], contributing to the quality and relevance of co-designed outcomes [42]. This aligns with the Design Thinking framework, specifically the ideate and prototyping phases. While co-production with people with aphasia is increasing, there is limited evidence on co-design in SLT, especially for motor speech disorders [21,32,43]. Therefore, this research continues to contribute to and develop the evidence base regarding co-design in SLT, particularly for people with dysarthria. This study is unique, as it is believed to be the first co-design study with PwPD who have speech and voice difficulties that co-designs solutions to problems experienced when using commercial VAT technology.

Participants described personal benefits of co-design, including gaining knowledge, social interaction, and feeling heard and validated, echoing previous co-production findings [32,44] and aligning with public involvement standards [45]. These benefits are particularly relevant for PwPD, who often experience reduced participation due to speech and voice issues [46], highlighting how co-design can empower participants. Power sharing and partnership can enhance engagement and lead to more patient-centered outcomes [47], and involving SaLTs may also improve future implementation of such tools into clinical practice [48]. In wider co-design research in SLT, participants with communication difficulties report improved confidence, motivation, and sense of well-being [27], and their involvement can lead to more and better-quality outcomes [43]. Overall, this demonstrates how co-production can allow participants, such as PwPD, to feel in control, empowered, and validated. For PwPD, this co-design study both physically and metaphorically provided them with a voice, building on current evidence. Despite this, wider research also acknowledges that relinquishing power in research can be challenging for researchers, requiring an active effort to make the co-design process truly collaborative [18].

Additionally, collaborator feedback highlighted the importance of skilled facilitation in enabling communication during workshops. Although evidence on co-design facilitation strategies for people with speech and voice difficulties is limited, facilitators used clinical experience and evidence-based strategies [32] to support PwPD. These included allowing preparation time before workshops, building rapport, giving extra time to speak, screen-sharing key points, regularly checking understanding, and summarizing discussions [43,49,50]. Such approaches are crucial for inclusive and accessible co-design. Some collaborators suggested improvements, such as offering more workshops and using multimedia formats to make tasks easier, which extends the evidence base on co-design with PwPD who have speech and voice difficulties. This balance of positive experiences and suggested improvements reflects the range of participants, lived experiences, and heterogeneous needs. Advantages and disadvantages of co-design methods should be evaluated from a range of perspectives to achieve a balance between the needs of a diverse group of PwPD.

Participants indicated that training for SaLTs in the therapeutic use of smart speakers for speech and voice difficulties was a priority. Wider research supports this finding, showing that education and guidance are required to support therapeutic adoption by SaLTs and PwPD [4,9-11], and that digital health interventions for older adults should include education in effective device use, digital literacy skills, and technical support throughout [51,52]. Tailored education and guidance may contribute to PwPD and SaLTs successfully adopting and using smart speakers to support speech and voice difficulties. As such, this study begins to advance understanding of how to support VAT adoption into clinical SLT practice. While smart speaker features make them valuable tools for chronic health management among older adults [53,54], older people in particular can struggle to comprehend the full range of smart speaker functions [11,54].

Guidance should clearly link device features to SLT goals to promote understanding and demonstrate how devices can help people achieve their SLT practice and related goals [55,56]. This may positively impact digital literacy for PwPD, supporting device adoption and regular use [56], again contributing to advances in knowledge regarding the clinical adoption of VAT. Similarly, SaLTs in our earlier research made several content suggestions for guidance to empower them to use VAT [9], including sample therapy plans, scripts, goal-setting frameworks, and evidence-based practice. However, this is the first study to collate these elements into an education and guidance prototype for SaLTs. Simplified guidance is particularly important, as clinicians often discontinue technologies they perceive as overly complex for clients [57]. Similar requirements for implementation guidance have been reported in SLT research using commercial technologies, such as virtual reality (VR) [19,58,59]. These studies highlight that therapeutic usage guides should promote the ease of use and usefulness of commercial technologies to support clinical adoption and provide opportunities to trial the devices. However, it is important to note that although commercial VR technology was used, the VR program itself was specifically created by researchers. This suggests that guidance must explain how smart speakers’ out-of-the-box “Alexa skills” are relevant to SLT, given that the commercial use of the technology is not intended to be therapeutic. Unlike custom VR programs, smart speakers are off-the-shelf products not originally designed for health care. Therefore, guidance must explicitly link commercial features to therapeutic aims and support clinicians in adapting features to individual client needs, ultimately contributing to the adoption of VAT into clinical practice.

Furthermore, privacy and data protection are significant barriers to the adoption of smart speakers [60]. Common concerns include the recording of conversations and data misuse, which can deter both clinicians and clients [51,61-63]. To address this, usage guides for PwPD should include clear, accessible privacy information, support informed consent, and clearly explain how devices handle user data [64]. Given SaLTs’ responsibility for safeguarding client data, guidance should map VAT’s GDPR compliance and potential risks to SLT governance policies, such as Data Protection Impact Assessments. This study, therefore, begins to answer questions posed by previous research [10] regarding how VAT may be implemented in accordance with clinical governance requirements. Previous findings highlight that many SaLTs lack clarity on which technologies meet governance and GDPR standards [57]. Reassuring both clinicians and PwPD about privacy may improve confidence and facilitate adoption [65]. Future evaluation of guidance acceptability and usability could apply frameworks such as the Technology Acceptance Model or Unified Theory of Acceptance and Use of Technology 2.

### Delivery

Participants suggested delivering training through Royal College of Speech and Language Therapy–led webinars, live demonstrations, and group sessions led by trained SaLTs. While previous research has not identified optimal delivery formats [4,10], this study provides new insights into practical implementation and contributes to the evidence base regarding the therapeutic use of VAT in SLT clinical practice. The literature indicates that older adults often prefer hands-on, task-based learning supported by written instructions [66,67]. A training program using VAT as a tool for activities of daily living with adults with cognitive communication disorders indicated a need for written, easy-to-follow instructions, with hands-on support to overcome low technological literacy [67]. Group-based workshops can offer a supportive, low-pressure environment for exploration and skill-building with in-person support [52]. This is particularly important for users with limited experience or confidence in digital tools.

Findings indicate that SaLTs are central to introducing and supporting smart speaker use in therapy. When clinicians demonstrate relevance and ease of use, PwPD may be more likely to adopt the technology [67]. By increasing perceived usefulness and reducing concerns, training can enhance performance expectancy and digital engagement. Additionally, previous research on integrating commercial technologies in SLT has highlighted the importance of multifaceted training approaches, including device trials, workshops, clinical manuals, and information technology support [58,68]. Additional methods, such as guided observation and co-delivered interventions, may be necessary to bridge the gap between knowledge and practice [58,69]. As such, this study begins to address gaps in knowledge regarding the implementation of VAT as a therapeutic tool for speech and voice difficulties associated with Parkinson’s disease.

Participants highlighted the need to develop SLT-specific features for smart speakers, designed for therapeutic use. For example, Cassano et al [70] described a SaLT building a custom skill. At the time of publication, at least three speech therapy Alexa skills existed: Speech Therapy Practice, Speech Device Practice, and Let’s Talk. Additionally, 2 further speech therapy skills were identified but are no longer publicly available on the Amazon Skills store: Speech Doctor, as discussed by Makin et al [71], and Speech Therapy by Cathal Killeen. Notably, Speech Therapy Practice is a live Alexa skill developed by a SaLT that enables people with aphasia to practice very basic words and phrases, such as colors, opposites, who/what questions, and yes/no questions. While this may potentially act as a starting point for SaLTs, the skill lacks applicability to practicing phrases and sentences and, in its current state, is unlikely to meet the speech practice needs of PwPD. To date, there are no specific Alexa skills for adults with Parkinson’s or targeting dysarthria, and our research highlights the potential for future development. Future research may seek to work with developers to create an Alexa skill for this population that can be used to support home practice of speech therapy exercises. Features may include prompts for loud, clear speech; increased feedback on volume and intelligibility with suggestions for improvement; the ability to monitor progress; visual displays and biofeedback; reminders to complete therapy tasks; and LSVT-style exercises with gamification [9,11]. Such features align with wider studies integrating technology into SLT and related areas, including apps using Google Glass [72], smart speaker–based physical activity interventions [73,74], and social engagement tools for people with disabilities [75]. Development platforms like Alexa Skills Kit and Alexa Blueprint may offer scalable, cost-effective options, enabling a focus on increasing motivation, engagement, and potential adherence to intervention programs. A curated hub of Alexa skills that can be used for SLT goals may also support clinical implementation. For example, Esquivel et al [76] developed a repository of Alexa skills and recommendations for people with disabilities, by people with disabilities. Future research may explore the acceptability of a speech therapy–specific Alexa skill and its implementation within clinical practice.

Given the commercial nature of smart speakers, it may be beneficial to first assess their current therapeutic value before creating bespoke skills. As our study focused on co-design processes and did not include a formal evaluation of intervention usability, effectiveness, or acceptability, future research may consider testing the current prototypes to determine real-world clinical impact and user outcomes. This study establishes the rationale for a future feasibility study to examine the effectiveness of VAT as a therapeutic tool for speech and voice difficulties in Parkinson’s disease. At the time of writing, no studies have been conducted in this area using commercial VAT. Emerging SLT research shows benefits for speech clarity in populations with intellectual disabilities and speech sound disorders [5,71], citing immediate rewards, spaced practice, enhanced autonomy, intrinsic motivation, and reduced social barriers as mechanisms of change in speech. However, these interventions do not follow established SLT intervention protocols [5,71]. Therefore, future studies should evaluate the effectiveness and usability of smart speakers for PwPD using principles of neuroplasticity and motor learning from SLT protocols, such as LSVT LOUD or Speak Out!

Despite this, the challenges surrounding the therapeutic use of smart speakers cannot be ignored. Smart speakers rely on evolving ASR models, a type of AI, which are continually being improved. ASR models can change without warning, presenting a risk to the reliability of baseline measurements and the measurement of therapy goals [77]. Furthermore, ASR errors are often higher than expected for dysarthric speech, speakers of minority languages, and those with regional accents [78-80]. Without clear and specific feedback on device or speech errors, both PwPD and SaLTs are left without information about where the “error” lies, whether it is speech- or device-related. These risks may reinforce maladaptive speech behaviors if speech practice is based on inconsistent or misleading responses from the device. It may also damage client motivation and confidence, with PwPD blaming themselves for technological errors. Research demonstrates that speakers can attribute ASR errors to themselves and link this to their sense of identity, including racial, regional, and locational identity [77]. To mitigate this lack of transparency, it is essential that SaLTs educate potential VAT users on strategies for adapting speech, managing frustration, and correctly interpreting VAT errors, as well as raising awareness of the limited ASR training on dysarthric speech and some minority or foreign languages [9,11]. This highlights the importance of a therapeutic usage manual for smart speakers for people with speech and voice difficulties and for SaLTs, as indicated in the current findings.

However, it should be noted that projects such as Voiceitt, Google Euphonia, and Project Relate aim to improve ASR accuracy in recognizing dysarthric speech, and Accessible Voice Interaction Technology for Aphasia (AVITA) aims to improve the accessibility of smart speakers [81], which may have the unintended consequence of limiting certain therapeutic applications of smart speakers in SLT. When smart speaker recognition is improved, speech difficulties no longer affect recognition, meaning all speech is easily recognized. This can be problematic, as speech that may not be intelligible in real life is recognized by devices. Consequently, this hampers therapeutic applications, because positive biofeedback provided by smart speakers does not reflect the speaker’s intelligibility to unfamiliar listeners in everyday contexts. Indeed, participants in this research indicated that future adaptations of smart speakers, outside of therapeutic contexts, should aim to better recognize dysarthric speech and regional accents. Future smart speaker designs may bridge the gap between standard out-of-the-box devices and fully customized skills. For example, smart speakers could allow users to set recognition thresholds, enabling both increased accessibility for users and therapeutic usage for clinicians. Given the rapid pace of innovation, continued review of emerging literature and technologies is recommended throughout the development and implementation stages.

### Limitations

This co-design study offered valuable insights into developing VAT tools for PwPD with speech and voice difficulties; however, limitations are evident.

Participants suggested an additional workshop between the ideation and prioritization phases, that is, between workshops A and B. A bridging session could have allowed more reflection and improved understanding, potentially leading to rankings that more accurately reflected lived experience. Furthermore, although recruitment was successful, there was some participant dropout between workshops A and B. This necessitated merging groups in workshop B, which may have influenced group dynamics and limited continuity of discussion.

Despite efforts to recruit a diverse group, the sample was small (n=20), and certain perspectives, such as those of people with advanced Parkinson’s or severe dysarthria, were underrepresented. This may limit the generalizability of the findings.

Finally, given the rapidly evolving technology landscape in AI and ASR, some recommendations may become outdated by the time of implementation. This includes changes in smart speaker capabilities, privacy policies, and integration with large language models (eg, AI conversational agents).

### Conclusions

This study highlights the value of co-designing smart speaker interventions with PwPD, carers, SaLTs, third sector representatives and technology and design experts to address challenges in using VAT for speech therapy. Using a participatory Design Thinking approach, user-centered solutions were generated to improve the accessibility, usability, and therapeutic potential of smart speakers.

Two prototypes were developed: (1) education and guidance for PwPD and SaLTs, and (2) speech therapy–specific smart speaker features.

The outputs balance commercial technology with clinical needs, focusing on privacy, troubleshooting, and feedback for home use, while reinforcing co-design as a powerful method for developing digital health tools. Co-design also ensured that interventions reflected lived experience and clinical insight, enhancing the likelihood of adoption and sustained use. This research strengthens the evidence for co-design in SLT and supports smart speakers as tools to enhance therapy access, promote self-management, and reduce pressure on SLT services. Future work should develop and evaluate these prototypes to assess their real-world impact and scalability.

## Appendix

Multimedia Appendix 1 Results condensed from workshop A.

Multimedia Appendix 2 Ranking to prototype creation.

Multimedia Appendix 3 Facilitator guidance.

Multimedia Appendix 4 Ranking results.

Multimedia Appendix 5 Prototype 1: education and guidance on the therapeutic use of smart speakers.

Multimedia Appendix 6 Prototype 2: developing new speech therapy–specific features for smart speakers.

## Abbreviations

AI: artificial intelligence
ASR: automatic speech recognition
GDPR: General Data Protection Regulation
LSVT: Lee Silverman Voice Treatment
PPI: patient and public involvement
PwPD: people with Parkinson’s disease
SaLT: speech and language therapist
SLT: speech and language therapy
VAT: voice-assisted technology
VR: virtual reality
