# Supplementary material for: Voice-Assisted Technology for People With Parkinson's Disease Experiencing Speech and Voice Difficulties: Co-Designing Solutions Using Design Thinking
Source: JMIR Rehabil Assist Technol. 2026 Feb 4;13:e84364. doi: 10.2196/84364 (PMC12917486; doi:10.2196/84364)
Supplement: Multimedia Appendix 1 [file rehab_v13i1e84364_app1.docx]

**Appendix 1 - Results condensed from workshop 1**

These ideas are presented as they appear within the workshops.

**How might we help people to understand smart speaker privacy and reduce their fears?**

| **Solution** |
| --- |
| Explaining to people what happens to their data e.g how / where data is stored, who owns it, where it is sold, can it be hacked, explaining why you get targeted ads, directing you to company policies |
| Myth busting with examples – explaining how privacy works, what does and does not happen to reduce fear, real world analogies of what to be aware of, what is important and what is just scare mongering |
| Outline what privacy settings are available and how to use them – e.g microphone control, clearing of history on the app, developing awareness |
| Smart speaker itself explains how to control privacy |
| Education that compares privacy settings across different popular commercial smart speakers |
| Why do smart speakers need your data? – help people to see the values alongside any risks e.g. if you share your birthday it means it can do X with, with explanations |
| Speaker itself reminding people you don’t have to share personal info (as a reminder during SLT practice) |
| Grading severities of GDPR – e.g what info you’re sharing which goes in which risk category |
| Clearer display of listening status / audible cues for when listening how started and stopped |
| Explain how to create safe spaces, free from listening in the home |
| Give people summaries – eg this week you used you speaker X times to practice your therapy, you have X sessions left this week before you see the SLT |
| Use a different trigger word instead of Alexa – to give people more control |
| Develop procedures for clinical use of smart speakers by SLTs – formal documentation, standard operating procedure eg data clearing, shared consent model for SLT and patient |
| Make IT / safety info that passed IT governance tests within NHS trusts for SLT – e.g. how to get it approved for use in clinic / NHS digital to verify and let SLTs know if it is recommended |

**How might we help people when smart speakers don’t work?**

| **Solution** |
| --- |
| Troubleshooting guide for SLTs - Is it the device / internet connection / volume problem etc – the SALT would need a checklist to ask this [device or person issue]. |
| PwPD keep a log e.g. when did it work / didn’t work e.g. didn’t work in the evening – this would help the therapist troubleshoot |
| Making listening time longer to prevent cutting people off - “*Wait for me to finish*” or “*Don’t do anything yet*” to pause listening time / bookend command like walkie talkie ‘over’ |
| Listen for quiet speech / rate and give feedback (eg whisper mode) (generic feedback not personalised) |
| Troubleshooting guide for people with Parkinson’s - eg why don’t you try speaking more loudly, why don’t you try slowing down , move closer, consider environmental impacts etc |
| Remind people that the point of using Alexa is to practice speech and to get better / frustration is normal with tech |
| Crib sheet of phrases and sentences for people to practice – with yes / no from device re its understanding. Begin with easy short sentences and build up to longer ones to build peoples confidence |
| Could include visual cues for volume and loudness or clarity of speech |
| Alexa with a screen, trascribing speech - real time for feedback so you can see what the device understands / what it doesn’t |
| Educate family on how to work around difficulties and when to stop use |
| Alternative input methods: Backup plan e.g. gesture/button press/companion app. Ties back to visual indications of listening vs not listening |
| Set up custom commands - eg ‘Christmas, santa is here’ or ‘good morning’ controls to reduce number of commands and errors |

**How might we help people to have a conversation with a smart speaker?**

| **Solution** |
| --- |
| LSVT through smart speaker – as an adjunct |
| Alexa conversation mode, follow up conversation and use talk mode – this would keep the mic open for longer to encourage natural back and forth for speech practice. |
| Read a poem/song/book, then Alexa reads with a back and forth – with prompts and supportive, does something exist / make something? |
| Have scripted conversations - practice conversation for GP, in the shop, how was your day or other scenarios that simulates real world conversation practice; needs to be conversation that users are comfortable with sharing? Could you request ‘Ask me questions about...” and prompts to help continue conversation |
| Program a routine – e.g good morning and good evening, E.g. how was your day, tell me more about that etc. Need empathy and be patient. |
| Build in a weekly reminder to prompt use (for therapy) |
| Prompt sheet – this is how you can speak and this is how you can have a conversation with it. Need visuals on the sheet. |
| Combine with screen eg transcribed speech showing |
| Explain to people why they should have a conversation with a smart speaker – why it would be useful for you. |
| Integrate prompts, and positive reinforcement (machine learning) similar to a real speech therapist - e.g. ‘could you speak a little louder?’, tell me more, well done you have finished practicing |
| Therapy game for smart speaker – to practice speech and voice |
| Eg Wikipedia of questions – eg question bank, list of questions tailored to needs (clarity) |
| Encourage integration into daily life - people to use it for recipes / joke / trivia |

**How might we help people to know what smart speakers can do?**

| **Solution** |
| --- |
| Education and catalogue of general uses and skills at set up – for PwPD |
| Help guide on tech - so people to know how to access uses and skills / how to set up features |
| Catalogue of speech therapy specific uses / skills – for SLTs |
| Peer support - ability to share solutions with one another / record and share examples of usage / advising other professionals what is out there |
| Training speech therapists – explicitly demonstrate how smart speakers work for speech and voice |
| Clear instructions on therapeutic use - how to use, with family members and carers, following up appointments and how to contact SLT etc; provide ideas or suggestions on what to say or ask / practice the words, key words |
| Loan bank of smart speakers to try out – could help with financial constraints so people could try out and see benefits for themselves |
| Webinar for SLTs with RCSLT - on how to use smart speakers as part of therapy |
| Daily tips features of Alexa – generate a workflow e.g. did you know I can help with your speech; how often do you want to practice today? |
| Get Trusted Tech group at Parkinson’s UK to do a review, and then a link to purchase it there – it might be more trusted, and people should be directed to this |
| Define what priorities and goals are for therapy (person centred) - match to specific tasks you can do with Alexa / split into categories |
| Integrating examples of use and where it could integrate into the user’s life |
| Feedback after using eg Nice work, you used a reminder for your medication, would you like to set a repeating one etc. |
| Link smart speaker to phone texting (radio voice notes) |
| Scope therapist understanding - to see what training needs are within teams, then tailor training to as many people as possible / enhance digital skills for therapists |

**How could we deliver this information?**

| **Solution** |
| --- |
| Training for SLTs – highlight how info is stored at the beginning, reassure about privacy |
| SLT’s delivering education and training to patients |
| Group based education |
| Information provision e.g visual aids, written user-friendly guide |

**How could smart speakers technology be adapted?**

| **Solution** |
| --- |
| Speak to manufacturers and get reassurances you can pass onto the patient re privacy |
| Smart speaker gives people info when they may need to share their data / disclaimer (just in privacy info) and how details can be updated |
| Develop requests to allow people to directly manage their data - e.g you can tell it how to delete something it just said e.g. wipe it |
| Ability to clear data with a command or request - forget what I just said, tell me my privacy settings, wipe data |
| Coloured rings to show 'private / not listening' 'listening but not saving' 'listening and saving' |
| Allow a command to “stop listening” |
| Can be set to recognise (NI) accent /  speech of people with speech and voice difficulties |
| Like Project relate app – listens to your speech and translates, it gets to know your pattern of speech and repeats back what you said, this might be helpful to build into Alexa |
| Change ‘Alexa’ to a more personalized name |
| Only answer to those trained to be heard (*more sensitive*) |
| Use a button / command for smart speaker to provide suggestions on what it isn’t doing, what you’re asking |
| A way to input data about specific needs |
| Ability to start again or get to rephrase things when you’re asking them |
| Knowing about the person’s routine eg meal / grandchildren. Responses are specific, to create rapport, smart speaker should know likes and dislikes |
| Defined with who you are speaking to, e.g. this conversation is not being recorded. |
| Real world triggers e.g. the person says ’im tired’ and Alexia says ’ Would you like me to’ |
