# Supplementary material for: Voice-Assisted Technology for People With Parkinson's Disease Experiencing Speech and Voice Difficulties: Co-Designing Solutions Using Design Thinking
Source: JMIR Rehabil Assist Technol. 2026 Feb 4;13:e84364. doi: 10.2196/84364 (PMC12917486; doi:10.2196/84364)
Supplement: Multimedia Appendix 2 [file rehab_v13i1e84364_app2.docx]

Appendix 2 – Showing ranking to prototype creation

| **Ranked Solution from Workshop 2** | **Coding** | **Categorization** | **Prototype** |
| --- | --- | --- | --- |
| Make IT / safety info that passed IT governance tests within NHS trusts for SLT – e.g. how to get it approved for use in clinic / NHS digital to verify and let SLTs know if it is recommended | Need more knowledge about privacy | Privacy of personal data | **Prototype 1:** Education and Guidance on therapeutic use of smart speakers |
| Give people summaries – eg this week you used you speaker X times to practice your therapy, you have X sessions left this week before you see the SLT | Need to increase motivation for speech practice | Increased feedback from smart speakers | **Prototype 2:** Developing new speech therapy specific features for smart speakers |
| Myth busting with examples – explaining how privacy works, what does and does not happen to reduce fear, real world analogies of what to be aware of, what is important and what is just scare mongering | Need more knowledge about privacy | Privacy of personal data | **Prototype 1:** Education and Guidance on therapeutic use of smart speakers |
| Smart speaker itself explains how to control privacy | Need more knowledge about privacy | Enhanced privacy features | **Prototype 2:** Developing new speech therapy specific features for smart speakers |
| Explaining to people what happens to their data e.g how / where data is stored, who owns it, where it is sold, can it be hacked, explaining why you get targeted ads, directing you to company policies | Need more knowledge about privacy | Privacy of personal data | **Prototype 1:** Education and Guidance on therapeutic use of smart speakers |
| Clearer display of listening status / audible cues for when listening how started and stopped (red light) | **Combined with ‘Explaining to people what happens to their data’** |  | **Prototype 2:** Developing new speech therapy specific features for smart speakers |
| Outline what privacy settings are available and how to use them – e.g microphone control, clearing of history on the app, developing awareness (red light) | Need more knowledge about privacy | Privacy of personal data | **Prototype 1:** Education and Guidance on therapeutic use of smart speakers |
| Troubleshooting guide for people with Parkinson’s - eg why don’t you try speaking more loudly, why don’t you try slowing down , move closer, consider environmental impacts etc | Navigating problems and difficulties | Troubleshooting guide on usage | **Prototype 1:** Education and Guidance on therapeutic use of smart speakers |
| Troubleshooting guide for SLTs - Is it the device / internet connection / volume problem etc – the SALT would need a checklist to ask this [device or person issue]. | Navigating problems and difficulties | Troubleshooting guide on usage | **Prototype 1:** Education and Guidance on therapeutic use of smart speakers |
| Making listening time longer to prevent cutting people off - “*Wait for me to finish*” or “*Don’t do anything yet*” to pause listening time / bookend command like walkie talkie ‘over’ | Need to adapt Alexa to better meet user needs | Extra new features | **Prototype 2:** Developing new speech therapy specific features for smart speakers |
| Remind people that the point of using Alexa is to practice speech and to get better / frustration is normal with tech | Navigating problems and difficulties  **(Combined with ‘Troubleshooting for PwPD’)** | Troubleshooting guide on usage | **Prototype 1:** Education and Guidance on therapeutic use of smart speakers |
| Could include visual cues for volume and loudness or clarity of speech | Biofeedback | Helping smart speakers reflect everyday conversations | **Prototype 2:** Developing new speech therapy specific features for smart speakers |
| Alternative input methods: Backup plan e.g. gesture/button press/companion app. | Touch accessibility - Catalogue of skills and standard smart speaker features | Therapeutic usage guide to target speech and voice | **Prototype 1:** Education and Guidance on therapeutic use of smart speakers |
| Alexa with a screen, transcribing speech - real time for feedback so you can see what the device understands / what it doesn’t | Live speech transcription | Increased feedback from smart speakers | **Prototype 2:** Developing new speech therapy specific features for smart speakers |
| Explain to people why they should have a conversation with a smart speaker – why it would be useful for you. | Explain rationale for using smart speakers | Therapeutic usage guide to target speech and voice | **Prototype 1:** Education and Guidance on therapeutic use of smart speakers |
| Have scripted conversations - practice conversation for GP, in the shop, how was your day or other scenarios that simulates real world conversation practice; | Routines or CHATGPT skills for conversation practice – **Catalogue of skills** | Therapeutic usage guide to target speech and voice | **Prototype 1:** Education and Guidance on therapeutic use of smart speakers |
| Program a routine – e.g good morning and good evening, E.g. how was your day, tell me more about that etc. | Routines or CHATGPT skills for conversation practice – **Catalogue of skills** | Therapeutic usage guide to target speech and voice | **Prototype 1:** Education and Guidance on therapeutic use of smart speakers |
| Alexa conversation mode, follow up conversation and use talk mode – this would keep the mic open for longer to encourage natural back and forth for speech practice. | Included adaptive settings – **Catalogue of skills and standard features** | Therapeutic usage guide to target speech and voice | **Prototype 1:** Education and Guidance on therapeutic use of smart speakers |
| Eg wikipedia of questions – eg question bank, list of questions tailored to needs (clarity) | Knowing what you can do with a smart speaker – **Catalogue of skills** | Therapeutic usage guide to target speech and voice | **Prototype 1:** Education and Guidance on therapeutic use of smart speakers |
| **LSVT through smart speaker –** as an adjunct | Need to adapt Alexa to better meet user needs | Create an Alexa skill for speech therapy | **Prototype 2:** Developing new speech therapy specific features for smart speakers |
| **Integrate prompts, and positive reinforcement (machine learning) similar to a real speech therapist -** e.g. ‘could you speak a little louder?’, tell me more, well done you have finished practicing | Need to adapt Alexa to better meet user needs | Increased feedback from smart speakers | **Prototype 2:** Developing new speech therapy specific features for smart speakers |
| **Therapy game for smart speake**r – to practice speech and voice | Need to adapt Alexa to better meet user needs | Create an Alexa skills for speech therapy | **Prototype 2:** Developing new speech therapy specific features for smart speakers |
| Webinar for SLTs with RCSLT - on how to use smart speakers as part of therapy | Training for SLTs | Delivery options for SLTs | **Prototype 1:** Education and Guidance on therapeutic use of smart speakers |
| Scope therapist understanding - to see what training needs are within teams, then tailor training to as many people as possible / enhance digital skills for therapists | Training for SLTs | Delivery options for SLTs | **Prototype 1:** Education and Guidance on therapeutic use of smart speakers |
| Catalogue of speech therapy specific uses / skills – for SLTs ; Education and catalogue of general uses and skills at set up – for PwPD ; Define what priorities and goals are for therapy (person centred) - match to specific tasks you can do with Alexa / split into categories ; Integrating examples of use and where it could integrate into the user’s life | Knowing what you can do with a smart speaker – **Catalogue of skills** | Therapeutic usage guide to target speech and voice | **Prototype 1:** Education and Guidance on therapeutic use of smart speakers |
| Help guide on tech - so people to know how to access uses and skills / how to set up features | Navigating problems and difficulties | Troubleshooting guide on usage | **Prototype 1:** Education and Guidance on therapeutic use of smart speakers |
| Training speech therapists – explicitly demonstrate how smart speakers work for speech and voice | Training for SLTs | Delivery options for SLTs | **Prototype 1:** Education and Guidance on therapeutic use of smart speakers |
| **Clear instructions on therapeutic use -** how to use, with family members and carers, following up appointments and how to contact SLT etc; provide ideas or suggestions on what to say or ask / practice the words, key word**s** | Clear instructions on therapeutic use | Therapeutic usage guide to target speech and voice | **Prototype 1:** Education and Guidance on therapeutic use of smart speakers |
| **Get Trusted Tech group at Parkinson’s UK to do a review, and then a link to purchase it there –** it might be more trusted, and people should be directed to this | Increasing trust in device | A review from Parkinson’s UK trusted Tech | **Prototype 2:** Developing new speech therapy specific features for smart speakers |
| Training for SLTs – highlight how info is stored at the beginning, reassure about privacy | Need more knowledge about privacy | Privacy of personal data | **Prototype 1:** Education and Guidance on therapeutic use of smart speakers |
| SLT’s delivering education and training to patients | SLTs deliver training and education for users | Delivery options for PwPD | **Prototype 1:** Education and Guidance on therapeutic use of smart speakers |
| Group based education for PwPD | SLTs deliver training and education for users | Delivery options for PwPD | **Prototype 1:** Education and Guidance on therapeutic use of smart speakers |
| Information provision for PwPD e.g visual aids, written user-friendly guide | Information provision | Delivery options for PwPD | **Prototype 1:** Education and Guidance on therapeutic use of smart speakers |
| Speak to manufacturers and get reassurances you can pass onto the patient re privacy / GDPR | Need more knowledge about privacy | Enhanced privacy features | **Prototype 2:** Developing new speech therapy specific features for smart speakers |
| Use a button / command for smart speaker to provide suggestions on what it isn’t doing what you’re asking | Need to adapt Alexa to better meet user needs | Increased feedback from smart speakers | **Prototype 2:** Developing new speech therapy specific features for smart speakers |
| Can be set to recognise NI accent / speech of people with speech and voice difficulties | At odds with therapeutic mechanism of VAT | At odds with therapeutic mechanism of VAT | Not included |
| Like Project relate app – listens to your speech and transcribes, it gets to know your pattern of speech and repeats back what you said. | Live speech transcription | Increased feedback from smart speakers | **Prototype 2:** Developing new speech therapy specific features for smart speakers |
| Allow a command to “stop listening” | Need to adapt Alexa to better meet user needs | Enhanced privacy features | **Prototype 2:** Developing new speech therapy specific features for smart speakers |
| Can AI be integrated? Eg Google Gemini - there is an ad on tv about conversing with your phone - can be moved into the smart speaker? Makes more human like, with 2-way conversation (1?) | Need to adapt Alexa to better meet user needs | Helping smart speakers reflect everyday conversations | **Prototype 2:** Developing new speech therapy specific features for smart speakers |
| Only answer to those trained to be heard (*more sensitive*) pros + cons | Make smart speakers more secure so others cannot speak to access info | Enhanced privacy features | **Prototype 2:** Developing new speech therapy specific features for smart speakers |
