# Supplementary material for: Voice-Assisted Technology for People With Parkinson's Disease Experiencing Speech and Voice Difficulties: Co-Designing Solutions Using Design Thinking
Source: JMIR Rehabil Assist Technol. 2026 Feb 4;13:e84364. doi: 10.2196/84364 (PMC12917486; doi:10.2196/84364)
Supplement: Multimedia Appendix 3 [file rehab_v13i1e84364_app3.docx]

**Appendix 4 - Facilitator Guidance**

**Workshop 1**

You will be assigned to a group of 4-5 participants. This may include people with Parkinson’s, carers, speech and language therapists, technology experts and staff from Parkinson’s UK.

Participants should be reminded that there are no silly ideas! The **focus** of the workshop is the **quantity of ideas,** rather than the quality or feasibility.

There will be a 5–10-minute introduction.

Breakout rooms will open. You will facilitate a small group to solve problem statements.

Once you are finished you will rejoin the main group and feedback on your ideas.

Each group will answer the 5 problem statements separately.

- **Each group will start with a different problem statement and rotate around until they have answered them all.** Here is the grouping and order of problem statements:

Group allocation link

- Each problem statement will have a separate OneDrive document link – therefore there will be 5 links. Facilitators should open each link, share their screen and type into each document. Please work on these offline, and then click continue to ensure the document updates.

Problem statement 1 link

Problem statement 2 link

Problem statement 3 link

Problem statement 4 link

Problem statement 5 link

- Other groups may already have typed on the document when you open it. **Do not show this on your screen - please scroll past.**
- Prompt participants by saying what would that look like or how could we do that?
- Spend **10 minutes** on each problem statement. 50 minutes total.

**Finishing up**

When smaller group work is finished, we will come together as larger group. Jodie will scan the documents and quickly summarise the main ideas.

**Facilitator Guidance Workshop 2**

You will be assigned to a group of 4-5 participants. This may include people with Parkinson’s, carers, speech and language therapists, technology experts and staff from Parkinson’s UK.

Participants should be reminded that there are no silly ideas! The **focus** of the workshop is deciding which solutions are important.

There will be a 5-minute introduction and welcome.

Breakout rooms will open. You will facilitate a small group to rank the top 5 solutions.

Once you are finished you should ask participants for their feedback on the co-design sessions.

You will then rejoin the main group and the sessions will close.

- There will be several solutions to each problem statement (created in the previous workshops), each group will have to rank 3 solution documents. For each document, rank the solutions 1-5.

Ranking 1 link

Ranking 2 link

Ranking 3 link

Ranking 4 link

Delivery link

Tech Adaptions link

- Each group will rank the solutions separately **and rotate around until they have answered them all.** Here is the grouping, order of working, and each group’s 3 solution documents. Please follow this.

Group allocation link

- Each solution will have a separate OneDrive document link – therefore there will be 3 links. Facilitators should open each link, share their screen and type into each document. Please work on these on the live onedrive version, using the desktop function and then click continue to ensure the document updates.
- Other groups may already have typed on the document when you open it. **Do not show this on your screen - please scroll past.**
- Spend **10 minutes** on each solution document. This is 30 minutes total. Please keep track of your own group's timings.

**Once you have finished the solutions, ask each group member for feedback. This is 1-2 lines, and record this in the last document you are working on.**

Ask questions like:

- What were your expectations of co-design before you started the workshops?
- Did you feel like you were heard, respected and your ideas were acted on?
- Can you describe how involved you felt in the co-design process? (Was your dignity maintained? Were you prepared appropriately? Did the online format work? Were the right people involved?)
- How do you feel about the results of the co-design process? Why?
- Did being involved in the project about using smart speaker for speech and voice difficulties have any impact on you? (If not, tell us why?)
