# Supplementary material for: Voice-Assisted Technology for People With Parkinson's Disease Experiencing Speech and Voice Difficulties: Co-Designing Solutions Using Design Thinking
Source: JMIR Rehabil Assist Technol. 2026 Feb 4;13:e84364. doi: 10.2196/84364 (PMC12917486; doi:10.2196/84364)
Supplement: Multimedia Appendix 4 [file rehab_v13i1e84364_app4.docx]

**Appendix 4 – Ranking results**

Each solution has between 4-7 solutions to rank. Numbers 1-5 show each groups top five priority solutions. Where both groups have ranked a solution, this highlights that they both perceived a solution to be important.

**1) How might we help people to understand smart speaker privacy and reduce their fears?**

| **Solution** | **Group 1 ranking** | **Group 3 ranking** |
| --- | --- | --- |
| Make IT / safety information that passed IT governance tests within NHS trusts for SLT – e.g. how to get it approved for use in clinic / NHS digital to verify and let SaLTs know if it is recommended | **1** | **5** |
| Give people summaries e.g. this week you used your speaker X times to practice your therapy; you have X sessions left this week before you see the SaLT | **2** | Not ranked |
| Myth busting with examples: Explain how privacy works, what does and does not happen to reduce fear, real world analogies of what to be aware of, what is important and what is just scare mongering | **3** | **2** |
| Smart speaker itself explains how to control privacy | **4** | Not ranked |
| Explain to people what happens to their data e.g. how / where data is stored, who owns it, where it is sold, can it be hacked, explaining why you get targeted ads, directing you to company policies | **5** | **1** |
| Clearer display of listening status / audible cues for when device is listening how started and stopped (red light) | Not ranked | **3** |
| Outline what privacy settings are available and how to use them – e.g. microphone control, clearing of history on the app, developing awareness (red light) | Not ranked | **4** |

**2) How might we help people when smart speakers don’t work?**

| **Solution** | **Group 1 ranking** | **Group 3 ranking** |
| --- | --- | --- |
| Troubleshooting guide for people with Parkinson’s e.g. why don’t you try speaking more loudly, why don’t you try slowing down, move closer etc. | **1** | **1** |
| Troubleshooting guide for SaLTs e.g. Is it the device or an internet connection problem. | **2** | **1** |
| Making listening time longer to prevent cutting people off - “*Wait for me to finish*” or “*Don’t do anything yet*” to pause listening time / bookend command like walkie talkie ‘over’ | **3** | Not ranked |
| Remind people that the point of using VAT is to practice speech and to get better / frustration is normal with tech | **4** | Not ranked |
| Could include visual cues for volume and loudness or clarity of speech | **5** | **4** |
| Alternative input methods e.g. gesture/button press/companion app. | Not ranked | **3** |
| Alexa with a screen, transcribing speech - real time for feedback so you can see what the device understands / what it doesn’t | Not ranked | **5** |

**3) How might we help people to have a conversation with a smart speaker?**

| **Solution** | **Group 2 ranking** | **Group 4 ranking** |
| --- | --- | --- |
| Explain to people why they should have a conversation with a smart speaker e.g. why it would be useful for you. | **1** | Not ranked |
| Have scripted conversations e.g. practice conversation for GP or other scenarios that simulate real world conversation practice. Could you request ‘Ask me questions about...” and prompts to help continue conversation | **2** | **2** |
| Program a routine e.g. good morning and good evening, how was your day etc. | **3** | **3** |
| Alexa conversation mode, follow up conversation and use talk mode – this would keep the mic open for longer to encourage natural back and forth for speech practice. | **4** | Not ranked |
| Wikipedia of questions e.g. question bank, list of questions tailored to needs (clarity) | **5** | Not ranked |
| LSVT through smart speaker as an adjunct | Not ranked | **1** |
| Integrate prompts, and positive reinforcement (machine learning) similar to a real speech therapist e.g. could you speak a little louder?’, tell me more, well done you have finished practicing | Not ranked | **4** |
| Therapy game for smart speakers to practice speech and voice | Not ranked | **5** |

**4) How might we help people to know what smart speakers can do?**

| **Solutions** | **Group 2 ranking** | **Group 4 ranking** |
| --- | --- | --- |
| Webinar for SaLTs with RCSLT on how to use smart speakers as part of therapy | **1** | **5** |
| Scope therapist understanding to see what training needs are within teams, then tailor training to as many people as possible / enhance digital skills for therapists | **2** | **3** |
| Catalogue of speech therapy specific uses / skills for SaLTs; Education and catalogue of general uses and skills at set up – for PwPD; Define what priorities and goals are for therapy (person centred) - match to specific tasks you can do with Alexa / split into categories; Integrating examples of use and where it could integrate into the user’s life | **3** | **2** |
| Help guide on tech - so people to know how to access uses and skills / how to set up features | **4** | Not ranked |
| Training speech therapists – explicitly demonstrate how smart speakers work for speech and voice | **5** | Not ranked |
| **Clear instructions on therapeutic use -** how to use, with family members and carers, following up appointments and how to contact SaLT etc.; provide ideas or suggestions on what to say or ask / practice the words, key words | Not ranked | **1** |
| **Get Trusted Tech group at Parkinson’s UK to do a review, and then a link to purchase it there –** it might be more trusted, and people should be directed to this | Not ranked | **4** |

**5) How could we deliver this information?**

| **Solution** | **Group 2 ranking** | **Group 4 ranking** |
| --- | --- | --- |
| Training for SaLTs – highlight how info is stored at the beginning, reassure about privacy | 1 | 3 |
| SaLT’s delivering education and training to patients | 2 | 2 |
| Group based education for PwPD | 4 | 1 |
| Information provision for PwPD e.g. visual aids, written user-friendly guide | 3 | 4 |

**6) How could smart speaker technology be adapted?**

| **Solution** | **Group 1 ranking** | **Group 3 ranking** |
| --- | --- | --- |
| Speak to manufacturers and get reassurances you can pass onto the patient re privacy / General Data Protection Regulation (GDPR) | **1** | Not ranked |
| Use a button / command for smart speaker to provide suggestions on what it isn’t doing what you’re asking | **2** | Not ranked |
| Can be set to recognise NI accent / speech of people with speech and voice difficulties | **3** | **2** |
| Like Project relate app – listens to your speech and transcribes, it gets to know your pattern of speech and repeats back what you said. | **4** | **4** |
| Allow a command to “stop listening” | **5** | **5** |
| Can AI be integrated? Eg Google Gemini - there is an ad on tv about conversing with your phone - can be moved into the smart speaker? Makes more human like, with 2-way conversation (1?) | Not ranked | **1** |
| Only answer to those trained to be heard (*more sensitive*) pros + cons | Not ranked | **4** |
