# Supplementary material for: Voice-Assisted Technology for People With Parkinson's Disease Experiencing Speech and Voice Difficulties: Co-Designing Solutions Using Design Thinking
Source: JMIR Rehabil Assist Technol. 2026 Feb 4;13:e84364. doi: 10.2196/84364 (PMC12917486; doi:10.2196/84364)
Supplement: Multimedia Appendix 5 [file rehab_v13i1e84364_app5.docx]

Prototype 1: education and guidance on the therapeutic use of smart speakers.

| 1. **Delivery methods for speech and language therapists (How)**  - **Training for speech and language therapists** - Scope therapist’s understanding to identify training needs within teams. - Explicitly demonstrate how smart speakers work for speech and voice. - Webinar with the Royal College of Speech and Language Therapists.  1. **Delivery options for people with Parkinson disease (How)**  - **Speech and language therapists deliver training and education** - Group-based education for people with Parkinson disease. - **Information provision** - User-friendly manual  1. **Content (What)**  - **Therapeutic usage guide—using smart speakers to help with speech and voice** - Explain the rationale for using smart speakers to help with conversation, volume, clarity, and intelligibility of speech, and how they can help. - Clear instructions on therapeutic use—also including screening, using with family, contacting speech and language therapists, and follow-up appointments. - A catalog of skills and standard smart speaker features that can be repurposed for speech therapy (eg, adaptive settings, touch accessibility with an Alexa Show, routines, ChatGPT skill for conversation practice, and example scripts). - Build a dictionary of words or questions to ask a smart speaker that are linked to specific speech needs. - **Extra information for speech and language therapists** - Define what priorities and goals are for therapy. - Match specific tasks with Alexa to specific clinical needs. - Examples of where smart speakers can be integrated into users’ lives and when to practice. - **Troubleshooting guide for speech and language therapists** - Checklist to determine causes of device error (eg, device, internet, or speech difficulty–related). - A help guide on smart speaker setup, including internet connectivity and how to access and use skills. - **Troubleshooting guide for people with Parkinson disease** - Suggestions for modifying speech to help recognition. For example, speak louder, speak slower, move closer, or consider environmental impacts. - A help guide on smart speaker setup, including internet connectivity and how to access and use skills. - Remind users that the purpose of Alexa practice is to improve speech, and frustration with technology is normal. - **Privacy** - Outline available privacy settings and how to use them. - Explaining what happens to patient data when using a smart speaker. - Myth-busting privacy speculation, including real-life examples. - Information on how smart speakers satisfy IT and governance requirements (speech and language therapy only). |
| --- |
