# Supplementary material for: Voice-Assisted Technology for People With Parkinson's Disease Experiencing Speech and Voice Difficulties: Co-Designing Solutions Using Design Thinking
Source: JMIR Rehabil Assist Technol. 2026 Feb 4;13:e84364. doi: 10.2196/84364 (PMC12917486; doi:10.2196/84364)
Supplement: Multimedia Appendix 6 [file rehab_v13i1e84364_app6.docx]

Prototype 2: developing new speech therapy–specific features for smart speakers.

| 1. **Delivery options**  - No delivery options were suggested by participants in co-design workshops.  1. **Contents (what)**  - Create an Alexa skill for speech therapy - Lee Silverman Voice Treatment through a smart speaker - Therapy game   This could include the following:   - **Increased feedback** - Visual cues on volume and clarity of speech via a screen. - Live speech transcription (like project relate). - Use of a command or a command that explains why Alexa is not responding in the desired way (speech and voice focused). - Provide visual summaries of weekly practice via a screen (eg, this week you used your speaker X times to practice your therapy; you have X sessions left this week before you see the speech and language therapist. - **Enhanced privacy features** - Clearer display of listening features (eg, cues or light). - Create a “stop listening” command. - Get reassurances of data privacy and eavesdropping from manufacturers. - A smart speaker only answers to the voice that it is trained to listen to. - **Helping smart speakers reflect everyday conversation** - Artificial intelligence integration for conversation (eg, like Google Gemini). - Making listening time longer to prevent cutting people off—“Wait for me to finish” or “Don’t do anything yet” to pause listening time. - **A review from Parkinson’s UK Trusted Tech** - Participants felt this would help to increase trust in the devices and their ability to help with speech and voice changes.   Participants also indicated that they would like smart speakers to better recognize dysarthric speech and Northern Irish accents. However, it is acknowledged that this is at odds with the therapeutic mechanism of voice-assisted technology. |
| --- |
